# Osteoperiosteal versus osteochondral for autologous transplantation in the treatment of large cystic osteochondral lesions of the talus

**DOI:** 10.1186/s10195-025-00818-1

**Published:** 2025-02-07

**Authors:** Lequan Liu, Jiangtao Jin, Jinping Pan, Huikang Guo, Sen Li, Jisheng Li, Zheng Zhang

**Affiliations:** Arthroplasty Dept, Jincheng General Hospital, 1st Kangping Road, Beishidian Area, Jincheng, 048006 Shanxi People’s Republic of China

**Keywords:** Osteochondral lesion, Talus, Osteoperiosteal, Transplantation

## Abstract

**Background:**

Osteochondral lesions of the talus (OLTs) with a large subchondral cyst have been shown to have inferior clinical outcomes after reparative techniques. Replacement techniques such as autologous osteoperiosteal transplantation (AOPT) and autologous osteochondral transplantation (AOCT) are indicated for large lesions. The aim of the study was to compare the short-term clinical and radiographic outcomes between patients undergoing AOPT and those undergoing AOCT for large cystic OLTs.

**Methods:**

Patients who underwent AOPT or AOCT for medial large cystic OLTs between May 2019 and June 2023 were retrospectively evaluated. According to their characteristics, 1:1 propensity‐score matching was performed, and 65 pairs of patients with ages ranging from 18 to 60 years old were recruited. Clinical outcomes were compared between both groups with the American Orthopedic Foot and Ankle Society (AOFAS) ankle-hindfoot score and Visual Analogue Scale (VAS). The Ankle Activity Score (AAS), time to return to sports activity (RTA), rate of return to sports level, complications, and results of a subjective evaluation were also collected. The integrity of subchondral bone and the quality of repaired cartilage were evaluated using the Magnetic Resonance Observation of Cartilage Repair Tissue (MOCART) score 12 months postoperatively. Second-look arthroscopy was performed 12 months postoperatively, and the cartilage repair was assessed with the criteria of the International Cartilage Repair Society (ICRS).

**Results:**

The within-group comparison showed significant improvements in pain severity and function in both groups post-treatment compared with pre-treatment. Between-group analysis, however, showed no significant statistical difference between groups in any of the variables for clinical and radiographic outcomes, except for donor-site morbidity of the AOPT group, which showed a better outcome compared to the AOCT group.

**Conclusions:**

In the treatment of large cystic OLTs, for patients with a chondral lesion of the patellofemoral joint that is unsuitable for AOCT, AOPT may be a safe and effective choice, with lower donor-site morbidity of the normal knee joint.

## Introduction

Osteochondral lesion of the talus (OLT) has been recognized as an increasingly common injury that usually occurs in acute ankle sprains, chronic ligament instability, and fractures [1, 2]. An OLT is an injury to cartilage and/or subchondral bone that may cause deep chronic ankle pain, swelling, stiffness, limited mobility, and even disability [3].

Several studies have reported that this lesion responds poorly to nonsurgical treatment and requires bone marrow stimulation or abrasion arthroplasty, with satisfactory clinical outcomes [4, 5]. However, Shimozono et al. found that subchondral cysts had a negative impact on clinical scores after surgery [6]. Although OLTs with small cysts could be treated effectively with microfracture or abrasion arthroplasty, lesions with a large subchondral cyst (with a diameter larger than 10 mm) may require replacement techniques such as autologous osteoperiosteal transplantation (AOPT) and autologous osteochondral transplantation (AOCT) [7, 8].

In the reconstruction of osteochondral defects, AOCT can provide bone and cartilage in the form of a plug and restore the weight-bearing ability of the talus [9]. AOCT has shown superior clinical outcomes when used to treat large cystic OLTs; however, donor-site morbidity of the normal knee is still a concerning complication. To date, according to published clinical studies of patients who received AOCT to treat large cystic OLTs, the percentage of patients with donor-site morbidity ranges from 0 to 54.5% [10, 11].

Recently, more and more surgeons have tried to repair large cystic OLTs with AOPT because of its low cost and the absence of donor-site morbidity in the knee [10]. In addition, Shi et al. have reported that AOPT shows favourable clinical outcomes and permits satisfactory incorporation of grafts into the tissue adjacent to this lesion [10]. However, to our knowledge, few studies have compared the clinical outcomes of AOPT and AOCT when they are used to treat OLTs with large cysts.

The primary purpose of this study was to investigate and compare the short-term clinical and radiographic outcomes of patients undergoing AOPT with those of patients undergoing AOCT for large cystic OLTs. We hypothesized that both procedures offered satisfactory results for the treatment of patients with large cystic OLTs and that donor-site morbidity of the knee occurred less frequently in the AOPT group than in the AOCT group.

## Methods

### Patient selection

This study was conducted in the authors’ department between May 2019 and June 2023. The study was reviewed and approved by the ethics committee of the authors’ hospital (LL2018110501). All participants with ages ranging from 18 to 60 years old were asked to sign a consent form before starting this study. Based on their characteristics, including sex, age, BMI, lesion size, lesion location, and second-look arthroscopy history, 1:1 propensity‐score matching was performed.

The inclusion criteria were as follows: (1) patients diagnosed with medial large cystic OLTs; (2) a lack of response to at least 3 months of nonsurgical treatment; (3) the diameter of the subchondral cyst was larger than 10 mm.

The exclusion criteria were as follows: (1) obvious structural malalignment (varus or valgus deformity of the ankle of more than 5°); (2) moderate and severe osteoarthritis; (3) systemic diseases, such as rheumatoid arthritis and gouty arthritis.

### Surgical intervention

All patients underwent diagnostic arthroscopy to diagnose a medial large cystic OLT after spinal anesthesia in the supine position. Moreover, the surface of the lesion was debrided. The centre of the defect was determined and drilled perpendicularly with a 2-mm pin. Then, the defect was drilled with a 4.5-mm cannulated bore until fat droplets from bone marrow were visualized. A harvester tube was used to create and enlarge the bone socket. The cyst and the sclerotic wall were debrided thoroughly by awls or small pins.

### AOPT group

**Fig. 1:**
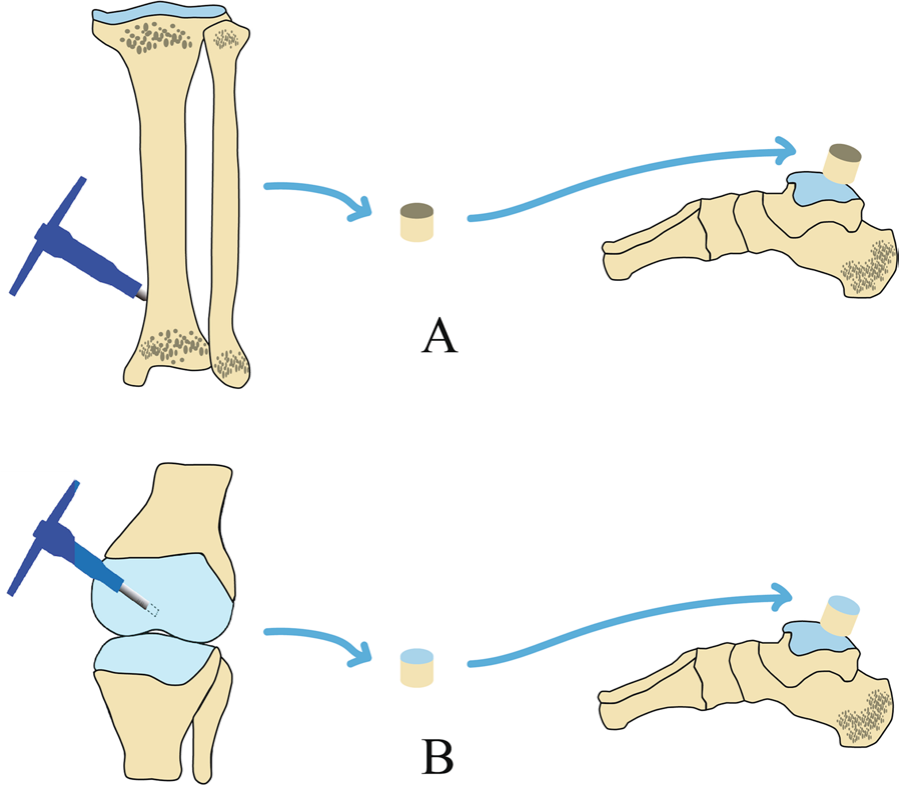
**A** The osteoperiosteal graft harvested from the distal tibia was implanted into the bone socket in the talus. **B** The osteochondral graft harvested from the non-weight-bearing portion of the ipsilateral medial femoral trochlea in the ipsilateral knee was implanted into the bone socket in the talus

Shown in Fig. 1A, a harvester tube was driven deeply and perpendicularly at a location approximately 1.5 cm above the osteotomy plane, and then the osteoperiosteal graft was harvested. Then, the graft was tapped into the cystic defect with the periosteum outward and without additional graft fixation. The graft was flush with the normal articular cartilage, and passive motion of the ankle joint was performed to confirm the stability of this graft. Then, the medial malleolus was reduced and fixed by cannulated screws driven up from the tip of the medial malleolus into the cancellous tibia bone. The anatomic reduction was confirmed by fluoroscopy [12].

### AOCT group

As shown in Fig. 1B, the osteochondral graft was harvested from the non-weight-bearing portion of the medial femoral trochlea in the ipsilateral knee, with the harvester tube driven perpendicularly. Then, the osteochondral graft was tapped into the bone socket with the periosteum outward and without additional fixation. The graft was flush with the normal articular cartilage. Passive motion of the ankle joint also was performed to confirm the stability of this graft. Then, the medial malleolus was reduced. Cannulated screws were fixed from the tip of the medial malleolus into the cancellous tibia bone, and the anatomic reduction was confirmed by fluoroscopy [13].

### Outcome measurements

Postoperative outcomes were assessed by the same investigator, who did not participate in the surgery. AOFAS score of each patient was evaluated preoperatively and at 12 months postoperatively [14]. The Visual Analogue Scale (VAS) consists of a 10-cm line on which the patient rates their pain in the range from 0 (indicating no pain) to 10 (representing the worst pain imaginable). The VAS was used preoperatively and at the 12-month follow-up [15]. An Ankle Activity Score (AAS) of ≥ 3 indicated that patients were able to walk on any uneven ground, and the time to return to sports activity (RTA) was considered to be the time taken for the patient to reach a minimum unlimited sports level of 3 on the AAS, which was recorded [16, 17]. The time taken to return to the previous sports level was defined as the postoperative time taken to reach the the same AAS as the pre-injury AAS, and this was also recorded. An overall subjective evaluation was also conducted at 12 months postoperatively. Patients were asked to grade the result of the procedure as either excellent (no symptoms), good (slightly annoying), fair (improved but with residual disability), or poor (symptoms unchanged or worsened). Excellent and good grades meant that the patient was satisfied with the surgery. Complications such as wound infection, donor-site morbidity, and wound numbness were evaluated and recorded during the follow-up. Magnetic resonance imaging (MRI) was performed 12 months postoperatively to compare radiographic outcomes between the two groups. The integrity of the subchondral bone and the quality of the repaired cartilage were evaluated using the Magnetic Resonance Observation of Cartilage Repair Tissue (MOCART; 0–100 points) score [18, 19]. Second-look arthroscopy was performed 12 months postoperatively, and the cartilage repair was assessed with the criteria of the International Cartilage Repair Society (ICRS) [20].

### Statistical analysis

A sample size calculation based on AOFAS scores from previous studies [using a two-tailed *α*: 0.05, *β*: 0.20 (power: 80%)] was conducted using G Power 3.1.9.7, considering an effect size = 1.33. Then we increased the estimated number by about 10% to ensure adequate power. It was determined that 65 participants would be required for each group. Shapiro–Wilk and Kolmogorov–Smirnov tests were used for testing the normality of the data distribution, and the results showed that all measured variables were normally distributed. Unpaired *t* tests and chi square were used to compare the characteristics of the subjects in the two groups. The Statistical Package for the Social Sciences computer program (version 20 for Windows; SPSS Inc., Chicago, IL, USA) was used for data analysis. *P* < 0.05 was considered significant.

## Results

### Patient demographics

**Fig. 2:**
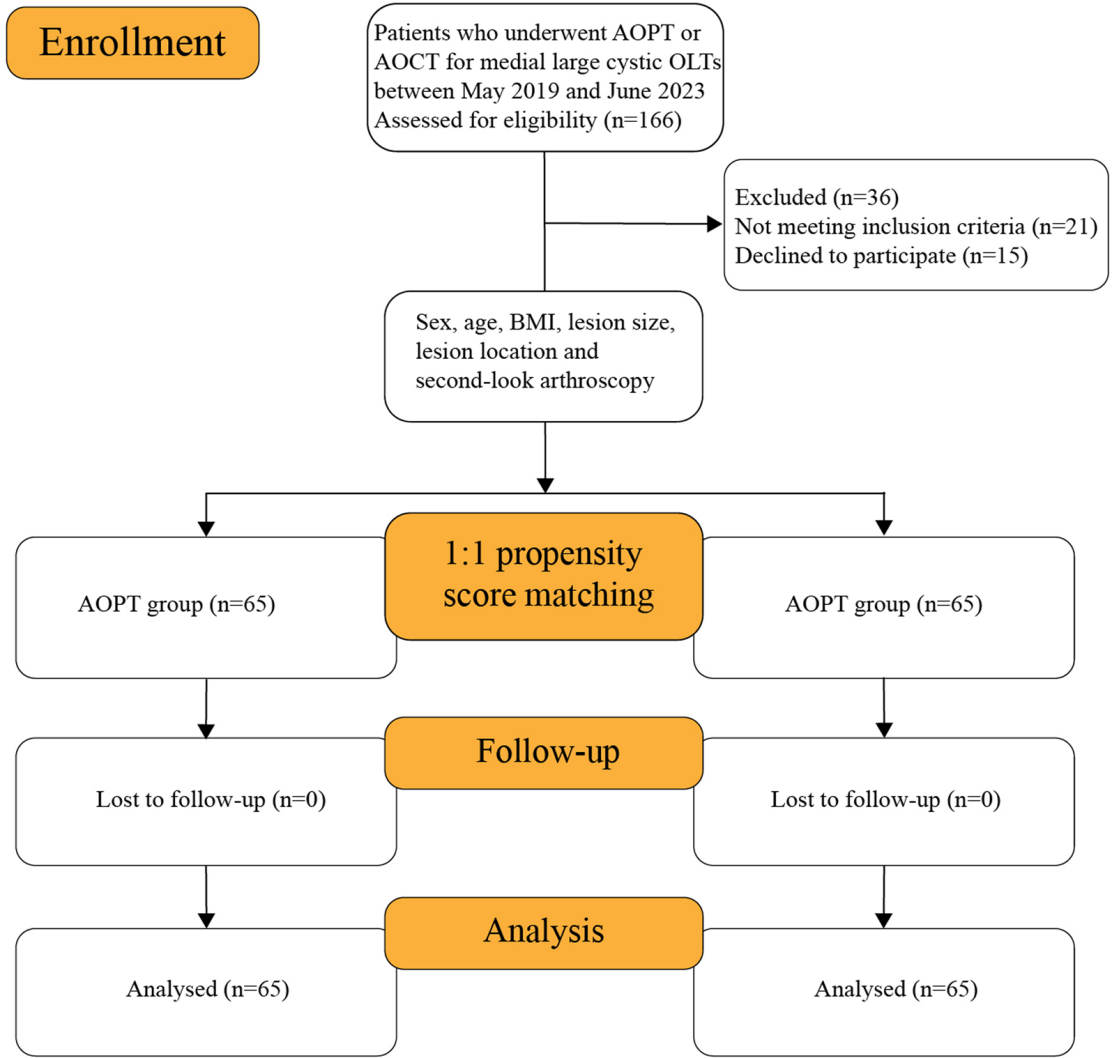
Flowchart for patient eligibility

Of the 166 patients assessed for eligibility, 21 patients did not meet the inclusion criteria and 15 patients refused to join the study (Fig. 2). The resulting study population consisted of 88 men and 42 women aged 18 to 60 years old with large cystic OLTs, who were randomly assigned to two equal groups. The two groups were comparable: they presented no significant difference in any of the demographic characteristics (Table 1).

**Table 1:** Comparison of clinical characteristics between the two groups

	AOPT group (*n* = 65)	AOCT group (*n* = 65)	*P* value^a^
Mean age ± SD, years	40.38 ± 7.42	40.76 ± 7.36	0.767
Male sex,* n *(%)	43 (66.15%)	45 (69.23%)	0.851
Mean BMI ± SD, kg/m^2^	20.68 ± 1.01	20.90 ± 1.02	0.221
Mean duration of symptoms ± SD, months	42.09 ± 6.66	41.28 ± 7.17	0.503
History of trauma,* n* (%)	50 (76.92%)	48 (73.85%)	0.839
Right talus, *n* (%)	35 (53.85%)	42 (64.62%)	0.284
Mean lesion size ± SD, mm			
Length	11.34 ± 1.68	11.44 ± 2.15	0.741
Width	10.19 ± 1.48	10.36 ± 1.57	0.540
Depth	9.77 ± 1.34	9.83 ± 1.17	0.765
Diameter	12.04 ± 1.34	12.33 ± 1.69	0.294
Lesion location, *n* (%)			0.534
Zone 1	16 (24.62%)	15 (23.08%)	
Zone 4	23 (35.38%)	29 (44.62%)	
Zone 7	26 (40.00%)	21 (32.30%)	

Data are reported as *n* (%) or mean ± SD. *BMI* body mass index in kg/m^2^

^a^Independent *t* test or chi-square test. The *P* values shown are for intergroup comparisons. Significance was accepted for *P* < 0.05

### Clinical outcomes

The within-group comparison showed significant improvement in the AOFAS score and VAS score in both groups post-treatment compared with pre-treatment. Compared with the AOCT group, there was no significant difference in postoperative AOFAS score, VAS score, AAS, RTA, rate of return to sports level, and the results of the subjective evaluation in the AOPT group. However, donor-site morbidity showed a better outcome in the AOPT group compared to the AOCT group (Table 2).

**Table 2:** Comparison of clinical outcomes between the two groups

	AOPT group (*n* = 65)	AOCT group (*n* = 65)	*P* value^a^
AOFAS score, mean ± SD			
Preoperative	71.14 ± 3.35	70.51 ± 3.22	0.276
Postoperative	91.29 ± 3.37	91.74 ± 3.16	0.438
*P* value (within)^b^	< 0.001	< 0.001	
VAS score, mean ± SD			
Preoperative	6.08 ± 0.85	6.05 ± 0.98	0.848
Postoperative	1.02 ± 0.80	1.15 ± 0.75	0.312
*P* value (within)^b^	< 0.001	< 0.001	
AAS, mean ± SD			
Pre-symptom	8.34 ± 1.03	8.23 ± 0.92	0.547
Preoperative	1.26 ± 0.69	1.18 ± 0.62	0.537
Postoperative	7.58 ± 0.95	7.34 ± 1.01	0.157
Mean RTA ± SD, months	6.35 ± 0.67	6.42 ± 0.68	0.605
Return to previous sports level, *n* (%)	31 (47.69%)	28 (76.92%)	0.725
Complication, *n* (%)			0.028
Donor-site morbidity	0 (0%)	6 (9.23%)	
Rate of satisfaction, *n* (%)	100 (100%)	100 (100%)	1.000
Subjective evaluation, *n* (%)			0.210
Excellent	43 (66.15%)	35 (53.85%)	
Good	22 (33.85%)	30 (46.15%)	
Fair	0 (0%)	0 (0%)	
Poor	0 (0%)	0 (0%)	

Data are reported as *n* (%) or mean ± SD

*AOFAS* American Orthopedic Foot and Ankle Society, *VAS* Visual Analogue Scale, *AAS* Ankle Activity Score, *RTA* time to return to sports activity

^a^Independent *t* test or chi-square test. The *P* values shown are for intergroup comparisons. Significance was accepted for *P* < 0.05

^b^Paired *t* test. The *P* values shown are for intragroup comparisons. Significance was accepted for *P* < 0.05

### Radiographic outcomes

All patients underwent an MRI scan at the final follow-up performed at 12 months postoperatively. As shown in Table 3, compared with the AOCT group, there was no significant difference in MOCART score, cysts on MRI, bone marrow oedema, and incorporation in the AOPT group.

**Table 3:** Comparison of radiologic outcomes between two groups

	AOPT group (*n* = 65)	AOCT group (*n* = 65)	*P* value^a^
MOCART score, mean ± SD	75.08 ± 5.86	76.85 ± 5.91	0.091
Cysts on MRI,* n* (%)			0.860
Disappeared	35 (53.85%)	37 (56.92%)	
Decreased	30 (46.15%)	28 (43.08%)	
Bone marrow oedema,* n* (%)			0.371
Yes	42 (64.62%)	36 (55.38%)	
No	23 (35.38%)	29 (44.62%)	
Incorporation,* n* (%)			0.857
Full	26 (40.00%)	25 (38.46%)	
Part	39 (60.00%)	40 (61.54%)	

Data are reported as *n* (%) or mean ± SD

*MOCART* the Magnetic Resonance Observation of Cartilage Repair Tissue scoring system, *MRI* magnetic resonance imaging

^a^Independent *t* test or chi-square test. The *P* values shown are for intergroup comparisons. Significance was accepted for *P* < 0.05

### Second-look arthroscopic outcomes

All patients were evaluated for second-look arthroscopic outcomes when the internal fixation screws that were used to fix the medial malleolus were removed at 12 months postoperatively. A shown in Table 4, compared with the AOCT group, there was no significant difference in ICRS score and ICRS repair category in the AOPT group.

**Table 4:** Comparison of second-look arthroscopic outcomes between the two groups

	AOPT group (*n* = 65)	AOCT group (*n* = 65)	*P* value^a^
ICRS visual score, mean ± SD	10.08 ± 0.94	10.32 ± 1.13	0.180
Initially grafted surface	4.20 ± 0.62	4.37 ± 0.63	0.123
Integration to border zone	3.52 ± 0.50	3.56 ± 0.50	0.727
Macroscopic appearance	2.37 ± 0.63	2.40 ± 0.60	0.671
ICRS repair category,* n* (%)			0.852
Grade I: normal	21 (32.31%)	22 (33.85%)	
Grade II: nearly normal	44 (67.69%)	43 (66.15%)	
Grade III: abnormal	0 (0%)	0 (0%)	
Grade IV: severely abnormal	0 (0%)	0 (0%)	

Data are reported as *n* (%) or mean ± SD

*ICRS* International Cartilage Repair Society

^a^Independent *t* test or chi-square test. The *P* values shown are for intergroup comparisons. Significance was accepted for *P* < 0.05

## Discussion

This study prospectively compared the outcomes of AOPT and AOCT as surgical options for large cystic OLTs. No significant statistical difference was observed between the AOPT and AOCT groups in any of the variables for clinical and radiographic outcomes except for donor-site morbidity, for which the AOPT group showed a better outcome compared to the AOCT group.

The importance of subchondral bone in the pathogenesis of lesions of the subchondral region of the OLT has been well discussed by Deng et al. [21]. They found that when trauma causes damage to the cartilage and subchondral bone plate, high-pressure liquid flows continuously into the subchondral bone, which induces osteolysis and subchondral cysts [21]. In addition, previous studies reported that subchondral cysts had a negative impact on clinical outcomes after surgery such as microfracture or abrasion arthroplasty [22, 23]. Hence, it is necessary for patients to undergo replacement techniques such as AOPT and AOCT in the treatment of OLTs with a large subchondral cyst (a diameter larger than 10 mm). We support the notion that the subchondral bone plate could play an important role in maintaining cartilage metabolism by supporting the normal ankle pressure and blocking the brunt of the continuous high-pressure articular liquid. Therefore, AOPT and AOCT achieve satisfactory outcomes for OLTs with large cystic lesions by reconstructing the normal subchondral bone plate.

In this study, sex, age, BMI, lesion size, lesion location, and second-look arthroscopy history were matched before enrolment in order to decrease the effect of potential confounders such as the degree of lesion chronicity and patients’ activity levels before surgery on outcomes. Symptoms and history of trauma were also compared before analyzing outcomes. In the present study, postoperative clinical and radiologic outcomes were examined in detail in both groups at approximately 12 months of follow-up. In the AOPT group, the AOFAS score significantly improved from a mean of 71.14 to 91.29, the VAS score significantly improved from a mean of 6.08 to 1.02, the AAS significantly improved from a mean of 1.26 to 7.58, the mean postoperative MOCART score was 75.08, and the mean ICRS score was 10.08 at second-look arthroscopy. In the AOCT group, the AOFAS score significantly improved from a mean of 70.51 to 91.74, the VAS score significantly improved from a mean of 6.05 to 1.15, the AAS significantly improved from a mean of 1.18 to 7.34, the mean postoperative MOCART score was 76.85, and the mean ICRS score was 10.32 at second-look arthroscopy. These results show that excellent clinical and radiologic outcomes were achieved when using these two techniques in the treatment of OLTs with large subchondral cysts, which is similar to previous research [7, 8, 10, 24].

Moreover, in this study, compared with the AOCT group, there was no significant statistical difference in postoperative AOFAS score, VAS score, RTA, rate of return to previous sports level, rate of satisfaction, MOCART score, and ICRS score in the AOCT group. We believe that both two techniques are simple, safe, and effective surgical procedures for the treatment of large cystic OLTs, providing effective graft replacement and joint pressure support. However, AOPT yielded a better outcome in donor-site morbidity compared to the AOCT group. In the AOCT group, the graft was harvested from the non-weight-bearing portion of the medial femoral trochlea in the ipsilateral knee, so patients with donor-site morbidity would feel symptomatic and experience pain when climbing stairs. We support the notion that friction of the patella and femoral trochlea during knee flexion may increase donor-site morbidity of the ipsilateral knee. In addition, graft harvesting can cause the release of intra-articular proinflammatory cytokines that can induce pain postoperatively, leading to a symptomatic knee, which has become a concerning shortcoming of AOCT [25].

Various studies have reported clinical outcomes of AOPT for the treatment of large cystic medial OLTs, and grafts were harvested from the ipsilateral anterior superior iliac spine [10, 24, 26–30]. However, in our study, the autografts in the AOPT group were taken from the medial tibia instead of the iliac crest, which is similar to Chen et al [12]. Harvesting autografts from the tibia could reduce surgical incisions, which could reduce the risk of wound infection and sciatic nerve injury.

Cao et al. [31] found that the periosteal bone column of a patient had grown well but that several small low‐density areas appeared on the surface of the bone column, and these areas showed a relatively minor improvement in terms of their postoperative evaluation indicators. These findings may be related to premature weight‐bearing by the patient or to a re‐sprained ankle following surgery [28]. However, in our study, all patients in both groups were immobilized in a short leg cast, there was no weight bearing on the affected limb for 6 weeks after surgery, and partial weight bearing was allowed 7 to 8 weeks after surgery. At 8 weeks after surgery, full weight bearing was allowed, after healing of the osteotomy was confirmed by X-ray. Hence, in our study, all patients in both groups indicated that they were satisfied with the surgery in their overall subjective evaluation because of the scientific postoperative rehabilitation.

It is important to consider the limitations of this study. The duration of the follow-up period in this study was limited to a specific timeframe, and the long-term effects of AOPT versus AOCT therapy were not evaluated. Therefore, we will follow up at 5 years postoperatively in the future. Moreover, future prospective multi-center studies with longer follow-up periods are needed to provide more comprehensive insights into the comparative effects of these two treatment approaches. In addition, the lack of blinding could have introduced observer bias. While our study demonstrated significant positive outcomes of AOPT, several questions remain unanswered, presenting avenues for future research. Because of the limitation of the sample, smokers and osteoporotic patients might also have been included in this study. Firstly, the long-term effects of AOPT versus AOCT on clinical outcomes need to be explored. The biomechanical and histological outcomes of AOPT versus AOCT in the treatment of large cystic OLTs also need to be examined. Future research that endeavours to address these unanswered questions will contribute to a deeper understanding of the therapeutic potential of AOPT and further optimize its application in clinical practice.

## Conclusion

In the treatment of large cystic OLTs, for patients with chondral lesions of the patellofemoral joint that are unsuitable for AOCT, AOPT may be a safe and effective choice with lower donor-site morbidity of the normal knee joint.

## Data Availability

No datasets were generated or analysed during the current study.

## Abbreviations

OLTs: Osteochondral lesions of the talus
AOPT: Autologous osteoperiosteal transplantation
AOCT: Autologous osteochondral transplantation
AOFAS: American Orthopedic Foot and Ankle Society
VAS: Visual Analogue Scale
AAS: Ankle Activity Score
MRI: Magnetic resonance imaging
ICRS: International Cartilage Repair Society
MOCART: Magnetic Resonance Observation of Cartilage Repair Tissue
RTA: Time to return to sports activity
BMI: Body mass index
